# Intelligent Structure Design of Learning Seats in University Smart Classroom under the Background of Intelligent Education

**DOI:** 10.1155/2022/7986426

**Published:** 2022-06-13

**Authors:** Yang Gao, Li Li

**Affiliations:** College of Materials Science and Technology, Beijing Forestry University, Beijing 100083, China

## Abstract

Under the background of intelligent education, the study chairs of intelligent classrooms in colleges and universities should reflect humanistic care and design and guide students to establish a healthy learning style. To realize the intelligent classroom learning, the intelligent seat can be applied to different sitting positions or different heights and weights. This paper designs a control system based on MC9S08DZ60 and realizes the communication between the control system and the mobile app by combining the Bluetooth module based on CSR8670. At the same time, this paper proposes an intelligent design that can adjust the seat height by voice. LD3320 speech recognition module is used for voice control, and a push rod motor with a rising speed of 7 mm/s is used to complete the lifting to meet the requirements of different sitting positions and different heights and weights. Then, the folding design of the intelligent seat for classroom learning is carried out. Infrared detectors are used at the armrests on both sides of the seat. When people have a tendency to sit down, they will speed up deployment. When a person leaves the seat, the seat will automatically retract. Finally, the objective evaluation method of pressure distribution test experiment is compared with the subjective scale evaluation of students, which verifies the effectiveness of the shape design of the chair in keeping students' healthy sitting posture for a long time.

## 1. Introduction

Under the background of intelligent education, it presents new opportunities and challenges for university education informationization. Universities must actively promote the development of educational management informationization, deepen the integration of information technology and education and teaching, innovate educational concepts and teaching models, promote the integration and sharing of educational management data, and improve fine management and scientific decision-making capability. Learning in the context of intelligent education is characterized by an autonomous learning process, interconnected learning methods, and intelligent learning support [1], resulting in a new intelligent learning paradigm. The premise and guarantee for achieving intelligent learning is to create a good intelligent learning environment. The main research contents of intelligent learning environment [2, 3] are how to fully utilize the new generation of information technology to support the realization of the core features of intelligent learning and how to design intelligent learning environment according to different learning occasions to support different activity modes.

Teaching smothering chair, also known as class chair, refers to the chair used by students in class, which is the most frequently used seat in campus life. It is not only the main category of school chairs but also the focus of educational furniture. It also reflects the related characteristics of smothering products and public services. A healthy chair is one that can ensure students' healthy sitting posture, and a healthy chair can maintain the health of lumbar vertebrae. In design, the shape of the chair is very important because a reasonable shape can maintain the normal physiological curve of lumbar vertebrae [4]. Literature [5] pointed out that if you keep sitting forward for a long time, it will cause great physiological pressure on the intervertebral disc, muscles, and ligaments. In general, it is generally believed that a good sitting position means sitting very straight, and the back support can make the back muscles support the trunk. If there is no back support, the time for the back muscles to support the trunk will be greatly shortened. Therefore, few people can keep their backs straight for a long time. There are two ways to solve this problem: either by providing a backrest or by increasing the angle between the torso and thighs. Literature [6–8] evaluates and analyzes the differences between the current human body sitting posture and healthy sitting posture, and then according to the relevant principles of sitting posture, discriminates six common sitting postures, which are: leaning right (left), leaning head, bending spine, leaning forward, and leaning back. These contents provide a theoretical basis for the study of sitting behavior. The algorithm of face, eyes, mouth, and shoulder detection has been improved, however, no specific product design scheme has been put forward, and the detection of the most sitting positions has been explored theoretically and experimentally. Literature [7], from the perspective of ergonomics, summarizes the main parameters of a healthy sitting posture for students. The angle of the body in a better sitting posture is 4 at 90 degrees, and the angle of the body in the best sitting posture is 2 at 90 degrees and 2 at 135 degrees. Literature [8] found that when the neck of human body bends more than 20 degrees, it will cause different degrees of musculoskeletal diseases.

In the case that the chairs in the modern market cannot meet the needs of students, the study has improved the design of the learning chairs in the intelligent classrooms of colleges and universities from the perspective of health. After repeated tests and experiments, various scientific and reasonable design indexes are constantly explored, and the design ideas and methods of intelligent structure of learning chairs in intelligent classrooms of colleges and universities based on human factors engineering are put forward.

## 2. Learning Seat Design Principles in Smart Classroom

### 2.1. Comfort Principle

In the intelligent classroom, the comfort of the chair has an impact on the learning experience. Human factors, seat factors, and human-seat interaction are the three types of factors that affect comfort. Gender, height, weight, and BMI are just a few examples of human factors. Individual preferences influence seat comfort [9]. Size, material, and appearance are all factors to consider when choosing a seat. The pressure distribution on the seat surface is related to seat factors, and the seat back should conform to the human spine curve [10]. Table and chair height ratios, as well as color matching, are important factors to consider. The height ratio of tables and chairs varies according to the user. The height of seats should be easily adjustable to meet the needs of the majority of users. Colors used in moderation can help reduce visual fatigue and provide a pleasant visual experience [11, 12].

### 2.2. Functional Principle

To cater to the intelligent classroom, tables and chairs should not be limited to the basic functions of sitting, writing, and receiving. The functional design of seats can be improved in many aspects, such as material, size, function, and color. In addition to the conventional physical improvement means, such as height adjustment, desktop tilt, roller addition, folding, or deformation of tables and chairs, we can also focus on technical improvement means, such as classroom interaction and resource sharing. For example, the application of Bluetooth transmission, virtual desktop technology, VR (Virtual Reality), AR (Augmented Reality), and other information technologies can optimize management, improve efficiency, facilitate data transmission, help students better integrate into the classroom, and improve students' participation and experience [13].

### 2.3. Interaction Principle

Wisdom class deviates from the traditional teaching model by emphasizing teacher-student interaction. We can consider the teaching mode and the realization of interactivity when designing seats for interactivity. There are four different types of teaching modes: the traditional teaching method, in which the teacher speaks and the students listen, is the first. The second type of discussion is group discussion, in which students are divided into groups of varying sizes. Thirdly, there are class meetings or other group gatherings that require everyone to sit together for deliberation, voting, and other activities [14, 15]. Fourth, to conduct activities in the classroom, tables and chairs must be removed. The implementation of interactivity in the smart classroom necessitates more technical means. Seats should be placed in a convenient and uniform manner, and they should be able to be adjusted as the class mode changes.

## 3. Intelligent Structure Design of Seat

### 3.1. Seat Shape Deduction

The design of the seat surface is based on the principle that different parts of the buttocks bear different pressures. The pressure is the greatest at the ischial tuberosity of the human body, gradually decreases to the periphery, and the pressure is the lowest at the thigh of the human body. The body pressure of the seat back is mainly distributed in the scapula and lumbar vertebrae of the back of the human body [16]. The supporting positions of these two parts are called “shoulder rest” and “waist rest.” The seat “lumbar support” is approximately between the third and fourth lumbar vertebrae, and the seat “shoulder support” is approximately between the fifth and sixth thoracic vertebrae. In the design of seats, “lumbar support” is very important.

This paper mainly focuses on the medium-sized seats. As the design of a seat cushion is the key part of the seat to maintain students' healthy sitting posture, three types of seat cushion are designed: I, II, and III. The edge of the I seat cushion is raised, and the ischial tuberosity is raised with emphasis, and the legs have certain support. The overall shape of the B seat cushion is sleek, mainly heightening the hips and thighs. Type III cushion is relatively square, only heightening the rear side of the buttocks. The purpose of heightening the two sides and the rear edge of the cushion is to fix and limit the buttocks and thighs of the user and to correct the sitting posture of the user using the above methods.

**Figure 1:**
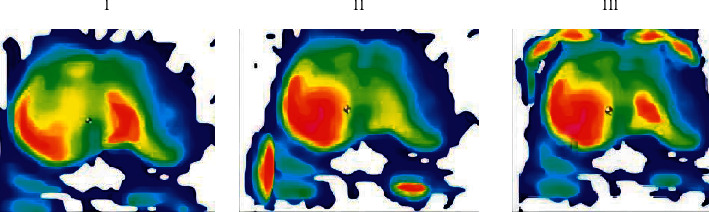
Hot zone map of three seat cushions.

As the cushion part is the key design part of the whole seat, firstly, the three preliminary designs are verified, and the most suitable cushion is selected. Then, the subsequent shape design and verification test are continued.  Figure 1 below shows the hot zone diagram of the sitting and pressing comparative experiment of the three seat cushions I, II, and III.

As shown in the comparison of the hot zone diagrams of the three seat cushions in the above figure, the hot zone of the thigh part of the II seat cushion is red, which indicates that the pressure value here is too high, the pressure on the thigh is relatively large, and the stress on the sitting surface is uneven, which is not conducive for maintaining a healthy sitting posture. There are many red areas on the rear side of the buttocks of Type III seat cushion, and the red area at the ischial tuberosity is large and mainly concentrated on the right side, which shows that the above areas exert great pressure on the human surface and are unevenly stressed, and the curved surface design of the sitting surface is reasonable, which does not meet the requirements of maintaining sitting posture. Although the pressure distribution of type I cushion is not ideal, the pressure distribution of type I cushion is relatively balanced among the three cushions. Therefore, type I cushion is selected as the initial seat shape, which provides a basis for the subsequent shape deduction and verification test.

### 3.2. Design and Composition of the Mechanical Structure of Intelligent Seat in Intelligent Classrooms

**Figure 2:**
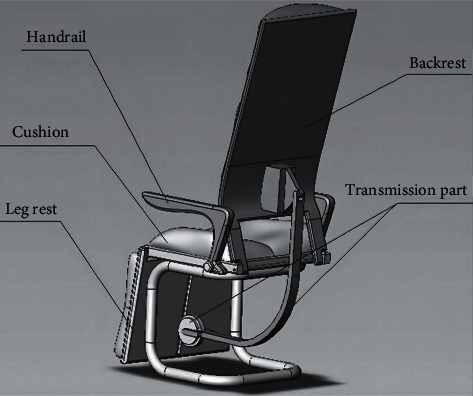
Learning 3D modeling of intelligent seat in intelligent classroom.

The mechanical structure of the intelligent seat for learning in the intelligent classroom is shown in Figure 2. The design inspiration comes from the seats with high utilization rate in the market, which mainly includes the floor bracket part, the control armrest part, the reversible seat cushion, the locking part, and the chair back part [17].

The floor support part is used to support the seat cushion, connect the seat cushion with the chair back, and make the whole chair be placed on the ground completely and reliably.

Back part: the design of the back part is made of wooden strips and is polished. Considering ergonomics and according to the data provided by references, the backrest and waist are designed [18] to make users have perfect riding experience as much as possible. At the same time, the main control board is placed at the back of the fixed backrest, which makes reasonable use of the space and facilitates the later program debugging and maintenance.

Control handrail part: used for placing fingerprint reading module, LED lights for displaying the status of attendees, and interactive buttons. It is connected with the main control board at the back of the backrest through the slot on the armrest, which ensures the stability of the flat cable and makes rational use of the space. Armrests allow users to place their arms to adjust their sitting posture, relieve fatigue, and improve their riding experience and comfort [19].

This part is inspired by the common reversible seat cushion and locking mechanism. This part, however, is equipped with a controlled electromagnetic locking device, unlike ordinary seats. The iron core in the locking device is in a pop-up state before the fingerprint signal is input. The iron core is inserted into the pin hole of the rotary seat cushion at this point, preventing the seat from being turned over, achieving the function of one person and one seat. The electromagnet is electrified after receiving the fingerprint unlocking signal, the iron core is adsorbed, the pin hole is unlocked, and the seat cushion can be turned over [20], completing the intelligent unlocking function.

### 3.3. Design of Intelligent Seat Control System in Intelligent Classroom

#### 3.3.1. Circuit Design

**Figure 3:**
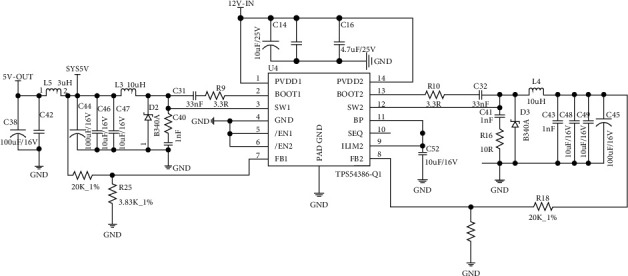
Circuit diagram of TPS54386 buck conversion.

To realize that the intelligent classroom learning intelligent seat can be used for different sitting positions or different heights and weights, this paper designs a control system for intelligent classroom learning intelligent seat, which realizes the above functions by controlling two stepping motors and one steering gear in the adjusting mechanism. There are many integrated circuits in the control system, which need to be powered by 24 V, 5 V, 3.3 V, and 1.5 V. TPS54386 is a converter with dual voltage outputs and nonstep down, with the maximum output current reaching 3 A, and the output voltage range can be selected between 0.8 V and 90% of the input voltage [21]. Therefore, TPS54386 is selected in this paper to step down 12 V to 5 V for position detection sensor, operational amplifier BA4558F, power amplifier NS4150, and steering gear and step down to 3.3 V for main processor MC9S08DZ60 and Bluetooth module CSR8670. The circuit diagram is shown in Figure 3.

To facilitate the operation, the control system adopts the way of mobile phone APP to realize the automatic adjustment of the intelligent seat in the intelligent classroom, and the mobile phone APP communicates with the control circuit board through the Bluetooth module [22]. With reference to the power consumption, transmission distance, transmission speed, and chip cost of different versions of Bluetooth, this paper uses the Bluetooth molding set BT86 based on CSR8670, which has a power rate equal to Class2 10, and it is packaged with the size of 20 mm (L) ∗ 10 mm (W) ∗ 1.8 mm (H). Its main features are as follows:

1. It fully supports Bluetooth V4.0 technical specification and is compatible with Bluetooth V5.0 technical specification.
2. 80 MHz RISC MCU and 80MIPS Kalimba DSP.
3. With integrated balun radio frequency module, the transmitting power of the radio frequency module is 10 dBm, and the receiving sensitivity is −90 dBm.
4. 16 Mb internal Flash memory, which can support external expansion to 64 Mb SPI flash memory.
5. Dual-channel ADC stereo audio decoder can support up to 6 microphone inputs.
6. The audio interface supports I2S, PCM, and SPDIF.
7. Support CSR's latest CVC noise reduction technology.

**Figure 4:**
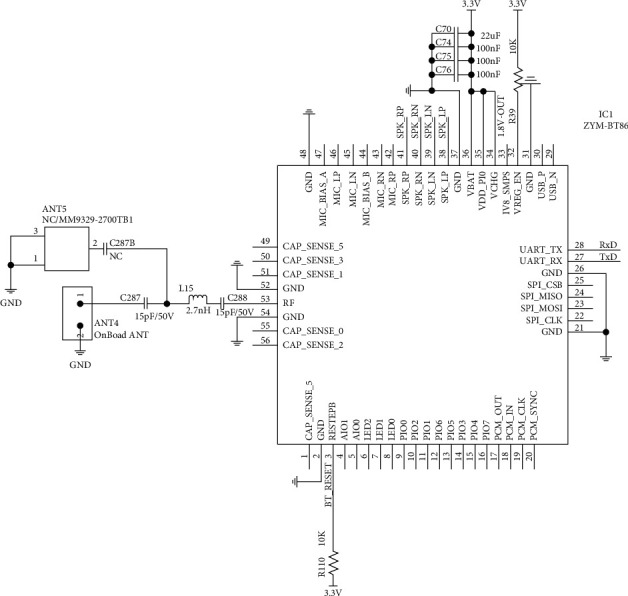
Circuit diagram of BT86 module.

According to the above characteristics, it can fully meet the functional requirements of intelligent classroom learning intelligent seat control system. The circuit schematic diagram of Bluetooth module BT86 based on CSR8670 is shown in Figure 4.

#### 3.3.2. Voice-Adjusted Seat Height Design

This paper proposes the design of intelligent seat height adjustment by voice based on the previous design to meet the needs of smart seats for various sitting positions, heights, and weights. The design of the voice-adjusted seat height in this section is based on STM32C8T6 single chip microcomputer, as well as the motor drive module, power supply module, and voice recognition module, which integrates the path planning algorithm and performs voice-controlled lifting.

**Figure 5:**
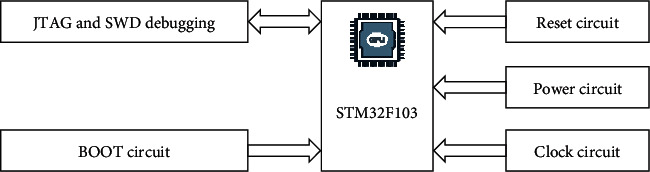
Structure diagram of STM32 core circuit.

*(1) Top Management System*. The STM32F103 series based on Cortex-M3 core should be used as the main control chip, with the highest working frequency of 72 MHz, including 32–512 kB Flash memory and 6–64 kB SRAM memory. There are 11 timers inside, with 3 12 bit ADCs, 2 IIC interfaces, and 5 USART interfaces and CAN interfaces. In addition, the chip is powered by a low voltage of 1.7∼3.6 V. The schematic diagram of the minimum system core is shown in Figure 5.

*(2) Lifting System*. The lifting is completed by adopting a putter motor with a rising speed of 7 mm/s, which is in line with the time difference from voice recognition to processing and issuing instructions by the voice module. The lifting is driven by DC, which transfers the horizontal rotation to the vertical lifting. By changing the positive and negative poles of the motor to ascend and descend, there is a travel limit switch at both ends of the push rod, and the telescopic rod will automatically lock when it reaches the bottom or reaches the top, thus avoiding the idling burning of the motor.

*(3) Voice Control Adjusts Seat Height*. LD3320 speech recognition module is used in speech control, and it has the function of double password control recognition: password mode + I/O control mode, which increases the recognition accuracy. The voice feature information we extracted is the input voice spectrum data. After setting the primary password, the next secondary password can be used for control operation. Furthermore, LD3320 supports speaker-independent speech recognition technology and does not need recording training. Speak specific speech recognition instructions (which have been entered in advance) through MIC microphone, carry out signal processing and judgment, and transmit the obtained results to STC slave control system for processing.

This module has a 5V TTL serial port and 16 I/O ports, which can communicate with external single-chip microcomputer, exchange information, and can also control relay and other equipment. Therefore, the voice recognition module is directly connected with the relay to control the lifting of the push rod motor.

*(4) Adaptive Weight Regulation*. The original purpose of the weight adjustment mechanism was to accommodate students of various weights in seats. When students of various weights sit in the seats, the upper and lower floors of the seats drop to varying degrees. Different students' weights can be quite different. Chinese students' weight can range from 45 to 100 kilograms. As a portion of a student's weight should be supported by their arms and legs, which do not act on the seat suspension system, 75 percent of them, according to experience, are supported by the suspension system. The seat's estimated dead weight is 10 kg. As a result, the possible variation range of the effective mass acting on the seat suspension system can be estimated to be 45–85 kg. Because each student's weight varies, the equivalent spring-loaded mass will vary as well. The compression amount of the vertical spring is different, and the length of the vertical spring is different when the seat suspension is in equilibrium. To make the seat suspension suitable for students of various weights, the lower end point of the vertical spring should change with the weight of the students, ensuring that the upper end point of the vertical spring is always in the same position when the seat suspension is balanced.

#### 3.3.3. Folding Design of Seat

The automatic folding seat with infrared sensor includes backrest, cushion, chain wheel, chain, limit switch, and control structure. The passive infrared sensor installed on the head of the seat senses whether someone exists, which has the following three functions [23]:

1. When someone approaches the seat, the seat will control the motor to rotate and slowly open the seat.
2. When a person stands temporarily and has not left the seat, the seat will not fold automatically.
3. When a person leaves, the seat will be folded slowly without perceiving the existence of the person.

**Figure 6:**
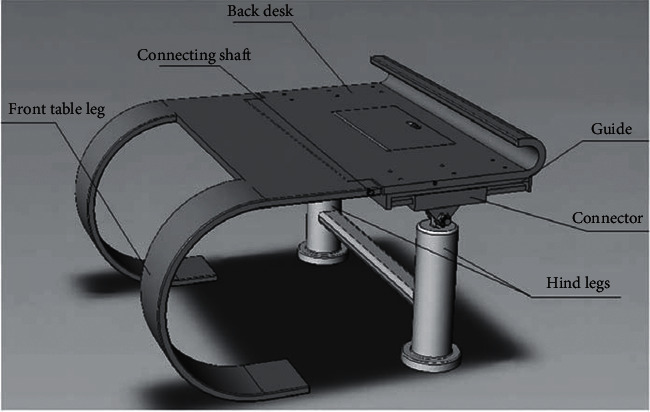
Structural design drawing of folding seat.

The structural design of the folding seat is shown in Figure 6 below.

**Figure 7:**
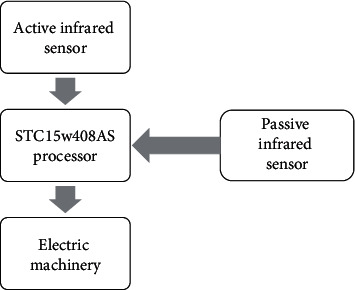
Hardware design block diagram.

The control structure comprises a controller, a brake, and a motor, wherein the controller is electrically connected with the brake and the motor, respectively. The motor is connected with the brake shaft through a coupling. The hardware of infrared foldable seat includes the following: core processor STC15w408AS, single chip microcomputer, active infrared sensor, passive infrared sensor, and motor. The hardware design block diagram is shown in Figure 7 below.

Select STC15w408AS single chip microcomputer, which integrates high-precision RC oscillation circuit and high-reliability reset circuit internally, so that the direct external crystal oscillator and reset circuit can be omitted, and the chip can work directly after being powered on, can output multichannel PWM to control the rotation of the motor, and sense whether someone approaches by reading the level change of the passive infrared sensor. By reading the level change of the active infrared sensor, we can sense whether someone has the tendency to sit down to speed up the rotation of the motor and open the seat.

Active infrared sensor has strong adaptability to ambient light. It has a pair of infrared transmitting and receiving tubes, which emit infrared rays with a certain frequency. When there are no obstacles in the transmitting and receiving tubes, the receiving tube receives the infrared rays emitted by the transmitting tubes, and the green indicator lights up. At the same time, the signal output interface outputs a digital signal and a low-level signal, and the sensitivity can be adjusted by a potentiometer knob.

The sensing module adopts a binary probe, the window of which is rectangular, and the binary is located at both ends in the longer direction. When the human body walks from left to right or from right to left, there is a difference in the time and distance when the infrared spectrum reaches the binary. The larger the difference, the more sensitive the sensing. When the human body walks from the front to the probe, from top to bottom, or from bottom to top, the binary cannot detect the change of infrared spectrum distance, and there is no difference. Hence, the sensing is insensitive or not working.

**Figure 8:**
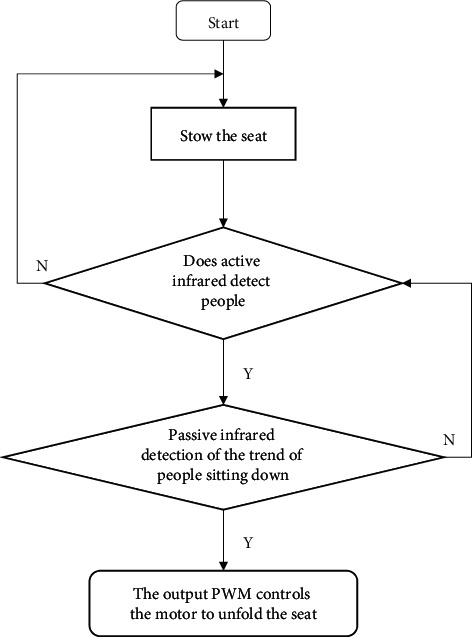
Flow chart.

The procedure of infrared automatic folding seat is shown in Figure 8.

1. Start running the program, initialize all parameters and functions, and judge whether the seat is folded.
2. The program senses whether someone exists by receiving the level change of the passive infrared sensor installed on the head of the seat. When someone approaches the seat, the level changes from high level to low level, and the MCU outputs PWM to control the motor to rotate and slowly open the seat.
3. The program judges whether people have a tendency to sit down by receiving the level change of the active infrared sensor, and if so, the MCU outputs PWM to control the motor to rotate and quickly deploy the seat.
4. When both the passive infrared sensor and the active infrared sensor are at high level, no user exists, and the seat is automatically folded.

## 4. Experimental Analysis

The medium seat is used as the research object for seat deduction in this paper. In this chapter, a comparison experiment is conducted between study seats in a university smart classroom and seats in a regular classroom to determine the effectiveness of the smart seat prototype designed in this paper in maintaining students' healthy sitting posture. Following the shape verification deduction of seat design, the design scheme of learning seat in the smart classroom of university is chosen to make physical products, and the prototype is made based on the short-time static test results. This chapter mainly verifies the two prototypes and assesses the subjective scale of the students. The effectiveness and rationality of the intelligent seat designed in this paper to maintain students' healthy sitting posture are confirmed through these two prototypes, and the effectiveness of the seat shape design and whether it meets the original purpose of designing the seat is confirmed through the pressure distribution experiment.

Experimentation: the subjects' body sizes were measured using a weight scale and a body size measuring ruler before the experiment began. Then, in a table, record the measured data. Students' classroom environment is simulated in the laboratory to make the experiment more scientific and reasonable. The students will sit in the standard sitting position while a computer plays the video of the teacher's lecture. The subject is asked about his or her subjective body feeling of seat comfort after the pressure distribution is measured. Complete the subject evaluation scale on a subjective scale.

**Figure 9:**
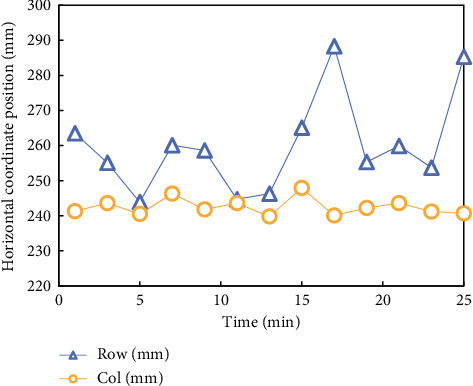
Line chart of the fluctuation of the central point position of common backrest pressure distribution.

**Figure 10:**
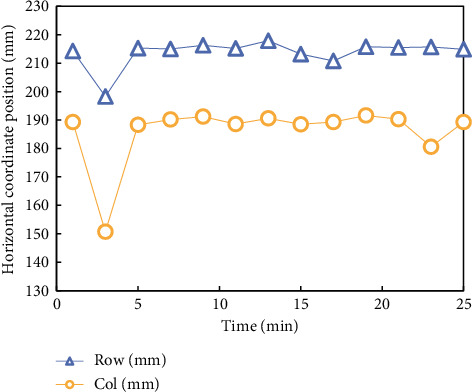
Line chart of the fluctuation of the central point position of pressure distribution on common chair surface.

After the experiment is completed, the experimental data are sorted and made into a data sample database, and then a set of data is randomly selected for comparative analysis according to the experimental comparison items. To observe the displacement of the subjects when using the seat for a long time more intuitively, a set of data is obtained through experiments, which is the plane displacement coordinate data of the pressure center points of the subjects' backs and buttocks, and the data are sorted out, as shown in Figures 9 and 10.

Figure 9 shows the fluctuation diagram of the pressure distribution center point of the common seat back, and Figure 10 shows the fluctuation diagram of the pressure distribution center point of the common seat surface. The broken line Row in the diagram represents the lateral displacement, and the broken line Col represents the longitudinal displacement.

When the subjects started the experiment, their sitting position and position shifted, and with the passage of time, the range of displacement became larger, especially the movement of the backrest was more obvious, which indicated that the ordinary seats had poor binding force on the subjects' sitting position and could not maintain their healthy sitting position.

**Figure 11:**
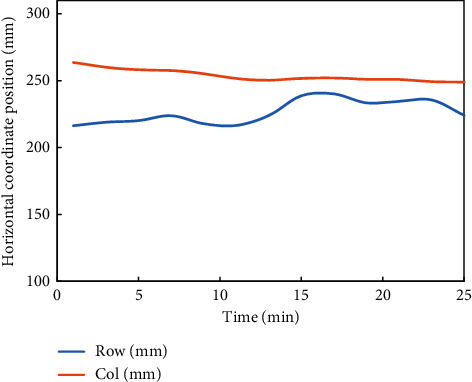
In this paper, a line chart of pressure distribution center point fluctuation of intelligent seat backrest is designed.

**Figure 12:**
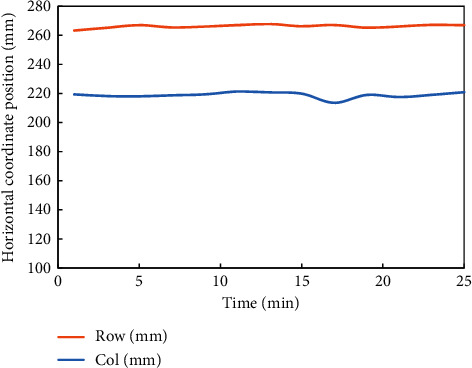
In this paper, the fluctuation line chart of the center point of pressure distribution on the seat surface of intelligent seat is designed.

Then, the pressure distribution of the intelligent seat designed in this paper is tested. To observe the displacement of the subjects when using the seat for a long time more intuitively, a set of data is obtained through experiments, which is the plane displacement coordinate data of the pressure center points of the subjects' backs and buttocks, and the data are sorted out, as shown in Figures 11 and 12.

It can be seen from the fluctuation of the pressure distribution center position in the above Figure 12 that the body displacement of the subjects is slight during the whole 25-minute test. These phenomena indicate that the intelligent seat designed in this paper can play a certain role in maintaining a healthy sitting posture and can maintain well the healthy sitting posture of the subjects. After the experiment, the subjective evaluation forms filled in by five students were sorted and analyzed. The statistical results are shown in Table 1.

From the comfort point of view, the comfort of ordinary seats is the worst. The intelligent seats designed in this paper feel better overall comfort. The shape design of the intelligent seat designed in this paper not only restricts the sitting behavior of students but also supports the back and waist to ensure a certain degree of comfort. Experiments show that the smart seat designed in this paper has reasonable comfort and can be used for a long time.

## 5. Conclusion

The research on the design of study seats in smart classrooms should be based on the construction and development status of smart classrooms, which can be considered from the perspectives of interactivity, functionality, and comfort and rely on Bluetooth, virtual desktop technology, VR, AR, and other information technologies to realize the automatic arrangement of seats, data transmission, classroom feedback, and efficient management, so that students can better integrate into the classroom and ensure teaching quality. Through the mechanical analysis of the chair, the intelligent student chair is designed with infrared folding structure, so that the backrest and cushion can move according to the rest needs of students at the same time. The antioverturning base ensures that the seat will not tilt backward and fall over when the students rest. The self-locking seat wheel can move freely when the seat is not stressed by a simple braking mechanism, and the seat wheel cannot move after the student sits down. In addition, the design of voice-adjusted seat height meets the needs of different sitting positions and different heights and weights. Compared with the seats in ordinary classrooms, the intelligent seats designed in this paper basically achieve the function of maintaining a healthy sitting posture, have a certain restraining effect on students' sitting posture, and provide a more reasonable pressure distribution. The shape design of the intelligent seat designed in this paper not only constrains the students' sitting behavior but also supports the back and waist, ensuring a certain degree of comfort. In the bionic modeling design, the size of the seat and the man-machine data are not sufficient. Hence, it is necessary to improve the data analysis of students' sitting posture. With the development of automation technology, all kinds of products serving human beings tend to be intelligent. Therefore, it is also the general trend to realize the intelligentization of learning seats.

## Figures and Tables

**Table 1:** Evaluation system of subjective scale for students' seat use feeling.

Classification of discomfort	No (1)	Slight (2)	Moderate (3)	Serious (4)	Very serious (5)
Seat type	Body parts				
	Shoulder	Back	Waist	Hip	Thighs
Ordinary seat	3	4	3	5	4
This paper designs an intelligent seat	1	2	1	2	1

## Data Availability

The data used to support the findings of this study are available from the corresponding author upon request.
